# Risk of tuberculosis transmission by children to healthcare workers – a comprehensive review

**DOI:** 10.3205/dgkh000439

**Published:** 2023-06-02

**Authors:** Roland Diel, Albert Nienhaus

**Affiliations:** 1 Institute for Epidemiology, University Medical Hospital Schleswig-Holstein, Kiel, Germany; 2 LungClinic Grosshansdorf, Airway Research Center North (ARCN), German Center for Lung Research (DZL), Grosshansdorf, Germany; 3 Institute for Health Service Research in Dermatology and Nursing (IVDP), University Medical Center Hamburg-Eppendorf, Hamburg, Germany; 4 Institution for Statutory Accident Insurance and Prevention in the Health and Welfare Services (BGW), Hamburg, Germany

**Keywords:** tuberculosis, children, healthcare workers, transmission, face masks

## Abstract

**Background:**

Healthcare workers (HCWs) are at increased risk of becoming infected with *M. tuberculosis* complex (Mtbc).

**Objective:**

To assess the magnitude of Mtbc transmission by children under the age of 15 years to HCW.

**Methods:**

Medline, Google Scholar and Cochrane library were searched to select primary studies in which a child was the presumed index case and exposed HCW were screened for latent TB infection (LTBI).

**Results:**

Of 4,702 abstracts, 15 original case reports covering 16 children with TB were identified. In sum, 1,395 HCW were contact persons and underwent testing. Ten of the studies reported TST conversion, amounting to 35 (2.9%) of the 1,228 HCW tested. In three of the TST-based and both of the studies that used IGRA testing, conversion was absent. 12 of the 15 studies (80%) reported exposure of HCW in neonatal intensive units (NICUs) to premature infants suffering from congenital pulmonary TB. One study including two infants addressed possible pulmonary Mtbc transmission in a general pediatric ward. Extrapulmonary transmission by aerosolized Mtbc was suggested in two patients, an infant with tuberculous peritonitis and a 12-year-old adolescent with pleurisy, and culture-confirmed only after the child had undergone video-assisted thoracoscopic surgery. Routine use of protective facemasks by HCW before exposure was not mentioned in any of the included studies.

**Conclusions:**

The results suggest that the risk of Mtbc transmission from children to HCW is low. Particular attention should be paid to infection risk during respiratory manipulations in NICUs. The consistent wearing of facemasks may further reduce the risk of Mtbc transmission.

## Zusammenfassung

**Hintergrund:**

Medizinisches Personal (MP) ist einem erhöhten Risiko ausgesetzt, sich mit *M. tuberculosis* *complex* (Mtbc) zu infizieren.

**Zielsetzung:**

Bewertung des Ausmaßes der Mtbc-Übertragung durch Kinder unter 15 Jahren auf MP.

**Methoden:**

Die Datenbanken Medline, Google Scholar und die Cochrane-Bibliothek wurden durchsucht, um Originalstudien zu finden, bei denen Kinder als mutmaßliche, an Tuberkulose (TB) erkrankte Personen beschrieben, und bei denen exponierte BiG auf das Vorliegen einer latenten Tuberkuloseinfektion (LTBI) untersucht wurden.

**Ergebnisse:**

Aus 4.702 Abstracts wurden 15 Originalstudien identifiziert, die 16 Kinder mit TB als mutmaßliche Infektionsquelle dokumentierten. Insgesamt waren 1.395 BiG Kontaktpersonen, die auch getestet wurden. 10 Studien berichteten über eine TST-Konversion bei 35 (2,9%) der 1.228 getesteten MP. In drei der TST-basierten Studien und in den beiden Studien, die IGRA-Tests verwendeten, gab es keine Konversion. Zwölf der 15 Studien (80%) berichteten über die Exposition von MP in neonatalen Intensivstationen durch Frühgeborene, die an angeborener Lungentuberkulose litten. Eine Studie mit zwei Säuglingen befasste sich mit einer möglichen pulmonalen Mtbc-Übertragung auf einer allgemeinen pädiatrischen Station. Bei zwei Patienten, einem Säugling mit tuberkulöser Peritonitis und einem 12-jährigen Jugendlichen mit Pleuritis wurde eine extrapulmonale Übertragung durch aerosolierte Mtbc vermutet, wobei die TB bei Letzterem erst im Rahmen einer videounterstützten Thorakoskopie kulturell bestätigt wurde. Die routinemäßige Verwendung von Gesichtsschutzmasken durch MP vor Exposition wurde in keiner der einbezogenen Studien erwähnt.

**Schlussfolgerungen:**

Die Ergebnisse unseres umfassenden Reviews deuten darauf hin, dass das Risiko einer Mtbc-Übertragung von Kindern auf MP gering ist. Besondere Aufmerksamkeit sollte dem Infektionsrisiko bei Atemwegsmanipulationen auf Neugeborenen-Stationen gewidmet werden. Das konsequente Tragen von Gesichtsmasken kann das Risiko einer Mtbc-Übertragung weiter verringern.

## Introduction

Tuberculosis (TB) is considered one of the most important occupational hazards for healthcare workers (HCW), especially for those who work in hospitals and medical offices where patients with yet-undiagnosed TB are admitted. According to the WHO, about 54% of healthcare workers in low- and middle-income countries carry latent TB infections (LTBI) [1], and in a systematic review that provided data on 30961 HCWs across 16 countries, LTBI prevalence among HCWs was more than twice that of the general population (odds ratio 2.27; 95% confidence interval 1.61–3.20) [2].

Although there is no doubt that HCW workers are generally at increased risk of LTBI due to their close and prolonged contact with TB patients, the risk of healthcare-associated transmission of *M. tuberculosis* complex (Mtbc) stems from a bundle of influencing factors [3]. The particular bundle facing HCWs dealing with TB-afflicted children in pediatric clinics and pediatricians’ offices, has to date been insufficiently investigated. While the transmission of Mtbc from HCW to children is well documented [4], [5], only single case reports are published from time to time regarding the risk of Mtbc transmission posed to HCWs by children under 15 years of age. For this reason, we conducted a comprehensive review of reports of MTBc transmission by children with a particular emphasis on risk mitigation, i.e., infection control practices, in the facilities examined.

## Methods

We performed a thorough literature review based on the PubMed electronic bibliographic database, the Cochrane library and Google Scholar. The following terms were used in Boolean searching: “pediatric,” “children,” “HCW”, “healthcare workers,” “transmission” and “tuberculosis”. Further selection followed according to the criteria below. Any studies published in peer-reviewed journals and that provided original data on suggested nosocomial Mtbc transmission to healthcare workers by children <15 years old were considered without restriction of publication date. Articles were excluded if their central theme diverged from or was not related to nosocomial transmission by children; reviews, guidelines and articles in languages other than English were also excluded. 

## Results

Out of 736 (Medline), 3,950 (Google Scholar), and 16 (Cochran Library) abstracts, 15 publications including a total of 16 children were found and analyzed in depth for the present article [6], [7], [8], [9], [10], [11], [12], [13], [14], [15], [16], [17], [18], [19], [20].

The studies from seven countries covered a publication period from 1991 to 2021. Most were from the USA (6/15, 40%), Australia, Canada, and Japan (2/15 each, 13.3%). Further source countries were Israel (1), South Korea (1) and Belgium (1) (see Table 1 (Tab. 1)). 

Of the 15 studies, 12 reported the exposure of healthcare workers in neonatal intensive units (NICUs) to premature infants born before the 37^th^ week of pregnancy and who suffered from congenital pulmonary TB. One study [8] addressed possible pulmonary Mtbc transmission in a general pediatric ward including two infants, aged five and 14 months, as well as a 12-year-old boy [18]. 

Extrapulmonary Mtbc transmission was the subject of two papers. In one, possible transmission by peritoneal dialysate disposed into the toilet over eight months in an initially 10-day-old boy with suspected tuberculous peritonitis [9] was discussed. In a similar case, a 12-year-old adolescent having undergone video-assistant thoracoscopic surgery (VATS) and subsequently diagnosed with culture-confirmed pleurisy, transmission to caregivers was suspected. 

A total of 1,395 HCW (hospital nurses, physiotherapists, anesthetists, or pediatricians) were identified as contact persons of 16 children and were tested at least once post-exposure for LTBI. With the exception of the studies by Bradford et al [14] and Tamura et al [19], HCW screening for LTBI was performed using the TST. Altogether and notwithstanding any possible bias of the formal results, 35/1,228 HCW (2.9%) who were tested with the TST were reported to be converters in 10 studies; in three studies, no TST conversion occurred. IGRA testing in the Bradford et al [14] and Tamura et al studies [19], also showed no probable conversion. No study reported that protective face masks had been worn routinely; the only mention of masks at all was by Saitoh et al [10] (“masks were worn after surprise tuberculosis was diagnosed”) and Khatami et al [18] (physiotherapists “often” do not wear masks). 

The requirements for undergoing contact screening as a HCW were quite variable across studies, ranging from “any contact” [7] to “direct” contact or specific time requirements (8 cumulative hours in the NICU [12], [14]). 

Two studies were unclear as to whether a parent was the true source of infection, rather than the infants being presumed as index cases. Rabalais et al. [8] emphazise that the transmission of MTB to three TST-positive HCW could also have been attributed to the infant’s father, who was suffering from TB as well. Crockett et al. [12] mention that their subject’s mother, although initially asymptomatic at the start of her son’s hospitalization, had infected the boy with her previously undetected pulmonary tuberculosis.

In the study by Matlow et al [9], aerosolization of tuberculous peritoneal dialysate was reported to have caused TST conversion in two nurses eight months later. Weakness of evidence leaves doubt as to whether the supposed index case was truly tuberculous peritonitis or a misdiagnosis based on laboratory error (contamination). The infant was TST-negative even after eight months, a source of infection could not be found, only one of four samples was culturally – but not microscopically TB positive, and – after therapy that had resulted in “clinical improvement” – no further peritoneal fluid samples were obtained to test for cultural conversion to negative.

In the study by Khatami et al [18], the 12-year-old male subject had no pulmonary TB, but only a culturally positive tuberculous pleurisy. A spread of Mtbc after VATS was blamed for creating a “confirmed” TST conversion in three physiotherapists during postoperative breathing exercises. Of note, those physiotherapists “often” did not wear face masks when working with/exposed to other patients as well.

In two of the three studies in which TST conversion occurred and in which the latency period between the last negative test and the date of conversion was reported, the latency was strikingly long: 15 and 33 months [9] or up to seven years [16]. Accordingly, the authors of the latter study [16] concluded that the three observed conversions could also be due to other tuberculosis patients. 

Mouchet et al. [13] point out that the conversion rate of 4.3% in their exposed HCW would not be statistically different from the conversion rate common for their entire hospital staff. However, no pertinent comparative data are found in the other studies.

## Discussion

In our review, despite an initially large number of citations on the topic, we located only very few original studies that addressed nosocomial transmission from children to HCW. Most of the 14 studies we found were from the field of neonatology. There, tuberculous aspirates from the respiratory tract during suction processes and concomitant manipulations, in connection with the extraordinarily close “face-to-face” contact of the caring hospital staff, may obviously be at least as infectious as the spread of droplet nuclei by coughing. To our surprise, however, with exception of the 12-year-old boy in Khatamy et al’s study [18], we could not find any publications on Mtbc transmission from children older than 14 months in the literature, although much older children are basically assumed to have a more vigorous cough and to be sputum-smear positive more often.

Because the tested members of the hospital staff did not appear to wear face masks before exposure, the low percentage of TST-positive HCW in our review cannot be explained by underreporting of true infectivity by protective measures. Nevertheless, although already low, the “true rate” of converters representing fresh infections in the included studies is likely to be much lower still because of individual methodologic weaknesses. In two of the 15 studies (13.3%), the source of Mtbc infection was just as likely to be a parent of the respective preterm infant as were the infants themselves. In the two studies where aerosolization of Mtbc was hypothesized after VATS of an adolescent with only culture-confirmed pleurisy [18] or during toilet disposal of tuberculous peritoneal fluid [9], the transmission process remains speculative. 

Finally, in another two of the 15 studies, the “confirmed” TST conversion due to exposure to a child suffering from TB must be questioned, as the interval between the last negative TST and the new positive test result is so long that the latent infections indicated by the conversions may have easily been caused by other hospital TB patients or by TB sufferers outside of the workplace. 

The attribution of TST conversions in the setting of ongoing exposure of HCW to Mtbc in hospitals or pediatric practices is problematic to begin with because, unlike IGRAS, PPD testing is subject to a booster phenomenon. Accordingly, freshly positive TST results that are interpreted as conversions could also be attributed to an Mtbc infection that occurred sometime in the past. In addition, false positive results in both serial TST testing of HCW and individual TST testing following contact may appear: This insight has been heavily re-inforced over the past 15 years, as the now widely-available IGRAS are proven to be far more specific than the TST in adults [21].

In one of the largest cohorts of HCW in a low-incidence setting, Dobler et al. [22] demonstrated that only 7% of TST HCW converters in the US had suspected tuberculosis exposure at the workplace, while the majority of TST converters (66%) had a negative IGRA test at the time of conversion. Also, in a study on French HCW [23] , the TST overestimated the prevalence of LTBI in this cohort, when TST results >10 mm were compared with the QuantiFERON-TB^®^ Gold in Tube test.

The above notwithstanding, the very low reported rate of TST conversions (2.3%) in HCW after contact with potentially infectious children is consistent with other recently published findings. With respect to Mtbc transmission by children suffering from infectious pulmonary TB, Diel et al. [24] investigated the literature published before August 2022 in a systematic review of genotyping studies that included all culture-confirmed transmissions to children and from children to other persons. Of 312 records retrieved, 39 studies including children aged <15 years offered epidemiological links between cluster members. In the 39 studies from 16 countries, 225 children were reported cluster members, of whom the overwhelming majority were infected by adults. Only three children, two of them younger than 10 years, were reported to be the definite source cases of eleven other children and one adult, who was not a HCW, with genotypically matched Mtbc isolates. Another genotyping study, a large-population-based prospective study from Hamburg, Germany, examined data from culture-confirmed TB patients diagnosed from January 1997 to December 2021. Here, whole-genome sequencing was applied for all samples [25]. Out of the total of 3,154 culture-confirmed TB cases, 52 children below the age of 15 years had pulmonary TB. Genotyping revealed that 35 of the 52 children were members of epidemiologically confirmed clusters and infected exclusively by adults, mostly household members, relatives or visitors to the refugee center accommodating the child’s family. Conversely, no HCW had acquired LTBI as contact persons of those children; moreover, during a mean observation period of 10.6 years, no secondary case that matched one cultured TB isolate from a child with pulmonary TB was found. 

## Conclusions

The results of our comprehensive review suggest that the risk of Mtbc transmission from children to HCW is very low. Particular attention should be paid to the risk of infection from respiratory manipulations in neonatal intensive care units. The strict wearing of face masks by HCW when dealing with unclear pulmonary infiltrates of newborns, children exposed to Mtbc in their family environment, or suspected maternal TB during pregnancy may further reduce the risk of Mtbc transmission. 

## Notes

### Competing interests

The authors declare that they have no competing interests.

### Author’s ORCID

The ORCID ID of Roland Diel is: 0000-0001-8304-7709

## Figures and Tables

**Table 1:**
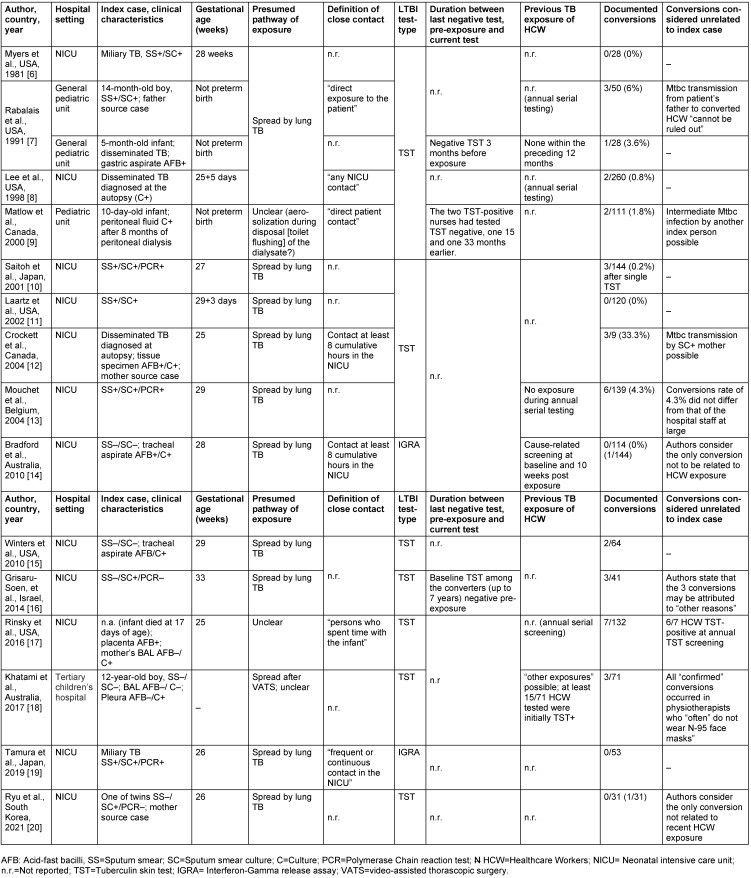
Case reports on healthcare workers exposed to children with TB disease
